# Ontogenetic shifts from social to experiential learning drive avian migration timing

**DOI:** 10.1038/s41467-021-27626-5

**Published:** 2021-12-16

**Authors:** Briana Abrahms, Claire S. Teitelbaum, Thomas Mueller, Sarah J. Converse

**Affiliations:** 1 Center for Ecosystem Sentinels, Department of Biology, University of Washington, Seattle, WA USA; 2 Odum School of Ecology, University of Georgia, Athens, GA USA; 3 Department of Biological Sciences, Goethe-University Frankfurt and Senckenberg Biodiversity and Climate Research Centre, Frankfurt, Germany; 4 U.S. Geological Survey, Washington Cooperative Fish and Wildlife Research Unit, School of Environmental and Forest Sciences & School of Aquatic and Fishery Sciences, University of Washington, Seattle, WA USA

**Keywords:** Animal migration, Behavioural ecology, Animal behaviour

## Abstract

Migrating animals may benefit from social or experiential learning, yet whether and how these learning processes interact or change over time to produce observed migration patterns remains unexplored. Using 16 years of satellite-tracking data from 105 reintroduced whooping cranes, we reveal an interplay between social and experiential learning in migration timing. Both processes dramatically improved individuals’ abilities to dynamically adjust their timing to track environmental conditions along the migration path. However, results revealed an ontogenetic shift in the dominant learning process, whereby subadult birds relied on social information, while mature birds primarily relied on experiential information. These results indicate that the adjustment of migration phenology in response to the environment is a learned skill that depends on both social context and individual age. Assessing how animals successfully learn to time migrations as environmental conditions change is critical for understanding intraspecific differences in migration patterns and for anticipating responses to global change.

## Abstract (web summary)

Learning from one’s own experience, and/or social learning from older individuals, could influence decision-making in migrating birds. Here the authors analyse 16 years of tracking data on whooping cranes to show that whether social or experiential learning is the dominant process in migration timing depends on life stage.

## Introduction

Billions of animals across diverse taxa migrate annually, with critical outcomes for population dynamics and ecosystem functioning^1^. How animals develop and achieve successful migration remains an enduring question. “Resource tracking” is widely recognized as a key mechanism underlying migration timing in a broad range of taxa^2^. The behavioral process of resource tracking allows migrating animals to enhance their energy gain by adjusting their timing during migration to keep pace with the progression of resource availability en route^3^. These adjustments in response to environmental conditions can prolong access to resources and increase individual performance^4^ and population persistence^5^. However, despite resource tracking’s ecological significance, its ontogeny, such as whether it is learned or innate, is poorly understood. Given that migratory species are among those most threatened by human-induced rapid environmental change^6^, understanding the mechanisms enabling animals to adjust their timing in response to environmental conditions is critical for determining how migratory species will be affected by global change.

Social learning, i.e. learning via transmission of information between conspecifics, and experiential learning, i.e. learning from one’s own past experiences, can both play important roles in migration. For species that migrate in groups, social learning can be critical to navigational success^7,8^. For long-lived species, individuals can also learn successful migration strategies through experiential learning^9–11^. Most migration studies have explored learning in the context of spatial navigation^7,8,11–13^, while very few studies have examined the role of learning in migration timing^9,10,14^. Furthermore, prior work has typically explored either social or experiential learning, yet in theory both can work in tandem or predominate at different life stages within an individual’s lifetime^15^. As a result, the relative roles of social versus experiential learning processes in migration, and how these roles may change by life stage, have not been investigated empirically.

Studying the ontogeny of migration timing in response to environmental conditions is challenging; it requires combining relatively high-resolution movement data collected over large spatiotemporal scales with individual life histories and data on social contexts across multiple life stages. Here we took advantage of a unique longitudinal dataset on reintroduced migratory whooping cranes (*Grus americana*) from a long-term monitoring program on all members of the population. This dataset provides not only migration tracks of individuals for over 15 years, but also robust knowledge of each individual’s social context during migration. We used this dataset to test the dual roles of social and experiential learning in the ontogeny of resource tracking behavior during migration.

**Fig. 1:**
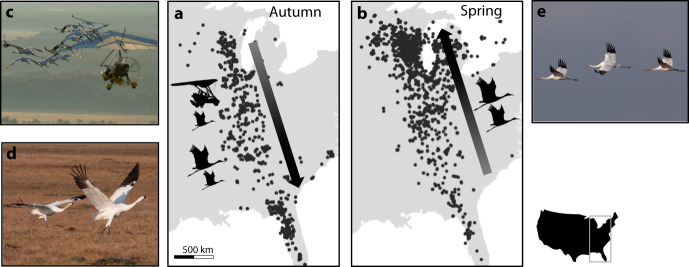
Whooping crane movement data and reintroduction program. Daily positions of 105 satellite-tracked whooping cranes aged 1–6 during (**a**) autumn and (**b**) spring. Reintroduced juveniles (<age 1) were trained to perform their first autumn migration by (**c**) ultralight aircraft or (**d**) conspecifics; spring migrations and subsequent autumn migrations were performed in mixed-age groups of conspecifics (**e**).

Whooping cranes are a long-lived (c. 30 years) endangered species and the world’s rarest crane (family Gruidae). Migratory populations were extirpated from eastern North America by the early twentieth century^16^. A captive-rearing program was initiated in the 1960s, and beginning in 2001, an eastern migratory population (EMP) was established, with three distinct rearing and migratory training methods used to accomplish the reintroduction^17^. In the first, used from 2001 to 2015, hand-reared juveniles (<age 1) were trained to migrate in autumn to their wintering range in Florida by following ultralight aircraft (Fig. 1). In the second, used beginning in 2005, hand-reared juveniles were allowed to follow older conspecifics during this first autumn migration. Finally, beginning in 2013, juveniles were parent-reared, rather than hand-reared, in captivity and were also allowed to follow older conspecifics on their first autumn migration. Subsequent migrations by juveniles, subadults (age 1), and adults (>age 1) were performed primarily in mixed-age groups of conspecifics^16^.

Long-term data from the reintroduction effort provides a detailed history of age and rearing/migratory training method for every individual in the population. In addition, all individuals were banded upon release and have been extensively monitored during their migrations via ground-based telemetry and visual observations, allowing complete identification of the composition of groups of birds as they migrate together. Further, of 504 total birds released and monitored, 105 birds aged 1–6 carried satellite-tracking devices between 2002 and 2018 to collect high-resolution relocation data during migrations.

Using these data, we built linear mixed models to test a series of hypotheses regarding environmental, social, and experiential influences on whooping crane migration phenology en route. To test whether cranes track resources during migration, we examined whether the latitudinal speed of satellite-tracked individuals as birds departed a given location was related to the environmental conditions encountered at that location. As whooping cranes are generalist foragers and have a strong association with vegetation greenness as a synoptic estimate of forage availability^18^, we examined responses to vegetation greenness measured by Normalized Difference Vegetation Index (NDVI)^19^. We additionally examined responses to snow depth, as snow or ice can cause birds to adjust the pace of their migration in response to lost foraging opportunities or conditions that otherwise threaten survival^20,21^. We hypothesized that cranes adapt their migration speed en route to environmental conditions, i.e., they track resources, and thus predicted that increasing snow depth would “push” birds south more quickly during autumn migrations, and increasing vegetation greenness would “pull” birds north more quickly during spring migrations.

To test the hypothesis that resource tracking behavior is socially learned, we examined two variables potentially influencing social information transfer: age of the oldest bird(s) in the group that an individual is migrating with (hereafter, “group age”) and rearing/training method (Supplementary Fig. 1). Prior work on this whooping crane population has demonstrated that collective knowledge in migratory groups, proxied by group age, improves spatial navigation in migration^7^. Thus, we predicted that birds would be more responsive to environmental conditions en route when migrating with older birds, and that the effect of group age would be strongest for subadults (age 1) as they perform their first independent migrations. Previous research has also shown lower behavioral plasticity in ultralight-trained cranes compared to conspecific-trained cranes in the first few years following release^13^. We therefore hypothesized that early learning would influence responsiveness to environmental conditions, and predicted that conspecific-trained birds would be more responsive to environmental conditions than ultralight-trained birds in autumn. We further predicted that, because of early social bonding of parent-reared birds to conspecifics, parent-reared birds would show greater responsiveness than hand-reared birds. To test the hypothesis that resource tracking behavior is also experientially learned, we examined the effect of individual age on response to environmental conditions. We predicted that responsiveness would increase with age, based on recent work that has shown that whooping cranes relocate their overwintering sites as they age based on prior experience^12,13^. Our findings reveal an ontogenetic shift in the learning of resource tracking, from a dominantly social learning process in early life to an experiential learning process as birds age.

## Results

### Effects of environmental conditions on migration timing

**Fig. 2:**
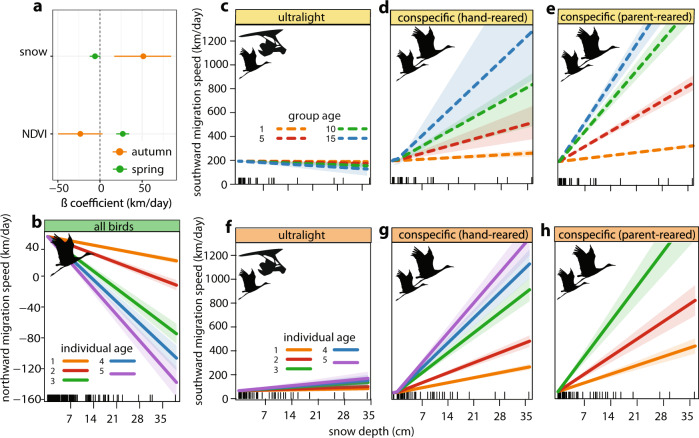
Environment, social learning, and experiential learning interact to affect latitudinal migration speed. **a** Main effect coefficients and 95% confidence intervals of the effect of snow depth and NDVI on latitudinal speed during autumn and spring migrations. Both variables are standardized for comparability. **b** Response lines for interactions between age and snow depth (cm) during spring migration. **c**–**e** Response lines for interactions between age of the oldest bird(s) in the migratory group (i.e., group age; dashed lines), training method, and snow depth (cm) for subadults in their first independent autumn migration. Response lines for group age = 1, 5, 10, and 15 years are shown for demonstration purposes. **f**–**h** Response lines for interactions between individual age (solid lines), training method, and snow depth (cm) for all satellite-tracked birds ages 1–5 years during autumn migration. Lines for ages 4 and 5 in parent-reared birds are not shown due to lack of data coverage. Individual and group age were modeled as continuous variables in all analyses. Rugplots at the bottom of each panel show data distributions; shading represents 95% confidence intervals.

We found clear support for resource tracking behavior during migration. Average durations of autumn and spring migrations were 17 days (range 2–122) and 27 days (range 3–162), respectively, with average daily speeds of 56 km/day (range 0–1216) in autumn and 48 km/day (range 0–858) in spring. As individuals encountered areas with high vegetation greenness, we found weak support for their movements southward slowing during autumn migration (*ß* = −23.3 km/day; 95% CI = −49.6–3.1 km/day), and strong support for their movements northward hastening during spring migration (*ß* = 27.1 km/day; 95% CI = 19.4–34.6 km/day) (Fig. 2a). In contrast, as individuals encountered areas with high snow depth, we found strong support for their movements quickening as they moved south during autumn migration (*ß* = 51.4 km/day; 95% CI = 17.0–84.4 km/day) and weak support for movements slowing as they moved north during spring migration (*ß* = −5.9 km/day; 95% CI = −12.2–0.4 km/day). Day of year had a negligible effect on migration speed in both seasons (autumn *ß* = −0.22 km/day; autumn 95% CI = −0.77–0.35 km/day; spring *ß* = −0.48 km/day; spring 95% CI = −0.71–0.24 km/day). Thus, cranes adjusted the rate of their latitudinal movements in response to the “push” and “pull” of environmental conditions. As snow had the largest effect (Fig. 2a), we report results below for snow, but all results were consistent for vegetation greenness (Supplementary Fig. 1). Betas reported are for standardized environmental covariates.

### Effects of social and experiential learning on migration timing

We found support for both social and experiential learning of resource tracking. However, results revealed an ontogenetic shift in the relative importance of social versus experiential learning processes. Subadults relied primarily on social learning: migrating with older birds significantly increased subadults’ responsiveness to environmental conditions during their first independent autumn migration, as indicated by a significant interaction between group age and environmental variables in the top-ranked model for subadults (*ß* = 29.1 km/day/year-of-group-age; 95% CIs = 15.8–42.1 km/day/year-of-group-age; Fig. 2d, e; Supplementary Table 2). In adults, an interaction with individual age supplanted group age in top-ranked models for both seasons (Supplementary Tables 3 and 4), such that older birds responded to conditions more strongly than younger individuals (autumn *ß* = 26.6 km/day/year-of-age; 95% CI = 19.4–34.6 km/day/year-of-age; spring *ß* = −3.2 km/day/year-of-age; 95% CI = −5.6– −0.6 km/day/year-of-age; Fig. 2b, g, h; Supplementary Fig. 2). All other candidate models had a ΔAIC > 2 compared to the top-ranked model for each season or life stage.

Importantly, how birds responded over time was also modulated by how they were initially trained to migrate to wintering grounds in autumn. An interaction with training method was retained in all top models (Supplementary Tables 2 and 4); ultralight-trained birds showed little response to environmental conditions independent of age during autumn (*ß* = 1.2 km/day/year-of-age; 95% CIs = −6.5–8.8 km/day/year-of-age), whereas hand-reared conspecific-trained birds (*ß* = 25.0 km/day/year-of-age; 95% CIs = 5.5–44.6 km/day/year-of-age) and parent-reared conspecific-trained birds (*ß* = 37.0 km/day/year-of-age; 95% CIs = −14.3–88.4 km/day/year-of-age) showed increasingly strong responses with age (Fig. 2c–h and Supplementary Fig. 2b). This continuum is consistent with the degree of intraspecific social bonding associated with each rearing/training method. Rearing/training method for training birds in their first autumn migration did not have a significant effect on behavior during spring migrations. Random effect variances for top models are reported in Supplementary Table 5.

### Effects of learning on exposure to environmental conditions

**Fig. 3:**
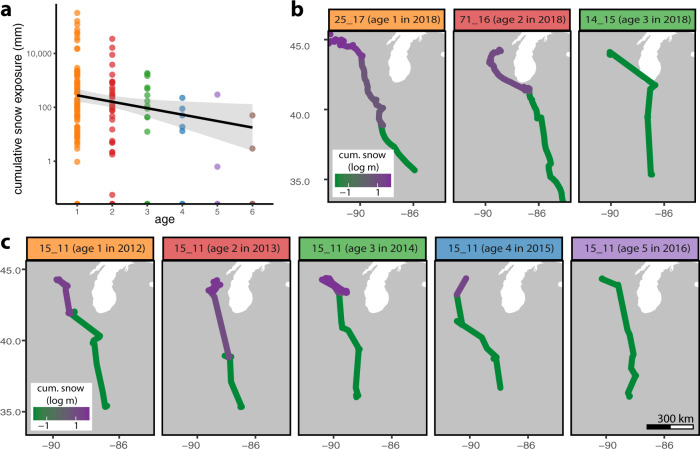
Cumulative snow experienced during migration decreases with age. **a** Total cumulative snow exposure experienced by conspecific-trained (hand- and parent-reared) birds during autumn and spring migrations by age (*n* = 242 migrations). Line and shading provide linear regression estimate and 95% confidence interval. **b** Cumulative snow experienced by three individuals ages 1, 2, and 3 years during spring migration in the year 2018. **c** Cumulative snow experienced by a single individual during spring migration as it aged from 1 to 5 years old.

Given the large effect of snow depth on movement rates (Fig. 2a), we examined birds’ cumulative exposure to snow over the course of their migrations as a function of age. Cumulative snow exposure provides a proxy for the foraging opportunities and energy expenditure that individuals experience during migration^20^. Even after controlling for interannual differences in winter severity, older individuals experienced significantly less cumulative exposure to snow over the course of their migration compared to younger individuals (Fig. 3a). Cumulative snow exposure during each migration decreased by nearly 2 m per year of age (*ß* = −1.8 m/year; 95% CIs = −2.0 to –1.5 m/year), with younger birds also exhibiting much greater variation in exposure. These differences could not be explained by age-related variation in departure dates at the start of each migration season (Supplementary Fig. 3). Thus, experiential learning enabled older individuals to tune their migratory movements to simultaneously track forage conditions and reduce cumulative snow exposure, which in turn can reduce energy expenditure during migration^20^.

## Discussion

Taken together, our analyses reveal that successful resource tracking during migration, i.e., the adjustments of timing en route in response to environmental conditions, in this long-lived social species is a product of social learning early in life combined with experiential learning over individuals’ lifetimes. Although theory predicts that experiential learning should dominate social learning in highly variable environments^22^, social information from older individuals can reliably produce adaptive behavior, due to their experiences of past environmental conditions^15^. Migrating with older birds allowed subadult cranes to better respond to environmental conditions encountered en route, indicating an important role of social learning in the ontogeny of behavioral plasticity during migration in this species. Further, how birds were initially trained to migrate south had a profound effect on plasticity in autumn migration timing, which provides a second line of evidence that social learning is especially important in early life. In adults, resource tracking was consistently stronger in older birds, indicating that experiential learning is an important modifier of migration phenology and that birds use their past experience to learn how to respond to environmental conditions.

Much recent research on avian migration has emphasized the timing of departure from and arrival to seasonal ranges rather than timing over the course of the migration, as examined here^23^. While the timing of departure or arrival has been frequently linked to age in birds, most studies only compare juveniles with adults^24^. Very few studies have examined departure or arrival timing in longitudinal studies lasting several years^9,10^, particularly as a function of the environment; by doing so, we show that social and experiential learning produce incremental improvements over an individual’s lifetime. We also demonstrate that learning is rapid in early years, as large differences in resource tracking abilities appeared within the first 3 years of individuals’ lives. Recently, resource tracking behavior has been shown to improve across generations at the population level in ungulates, suggesting that learning should occur at the individual level within a generation^14^. Here we provide empirical evidence that phenological adjustments in response to environmental conditions *during* migration—not just at its endpoints—can be learned, and that this learning depends on an interplay of social and experiential processes. Thus, our study provides an empirically supported mechanism in long-lived and/or social species that enables cultural transmission of resource-tracking behavior across generations. Furthermore, resource-tracking behavior is not limited to migratory species; many mobile consumers move to track phenological variation of resource across spaces, including nomadic, range-resident, and short- and long-distance migratory species^2^. Therefore, our results on the role of learning in resource tracking are likely to be applicable to a range of species beyond classical migrants.

Our findings add to a growing body of literature on the role of age and life stage on behavioral flexibility and innovation in free-ranging animals. In many species, the development of new or more flexible behaviors increases with individual age. This phenomenon is most likely to occur in long-lived species, as in our study. For example, elk learn over the course of their lives to adjust their habitat selection during hunting seasons in order to avoid hunters^25^. Across primate species, adults also are more likely to develop new foraging behaviors than juveniles or adolescents^26^. Greater behavioral flexibility and innovation in older individuals may be because these traits often build on other skills gained through individual experience and competency^26^. Such behavioral flexibility in older individuals can benefit whole groups or populations; in killer whales, for instance, the oldest females lead group movements to novel food sources in times of food scarcity^27^, and in whooping cranes, older birds established new overwintering sites that subsequently led to a population-level winter range shift^12^. However, in other species and contexts, younger individuals have shown much greater capacity for learning and flexibility in behaviors than adults, which then become solidified later in life^28^. This has important implications for adaptation to global change in those species, as the pace of behavioral adaptation will be limited by generation or maturation times; for example, in black-tailed godwits, population-level advancement in spring arrival timing was necessarily driven by new recruits, as arrival timing of individual birds remained consistent over time^29^. Our study indicates that different learning processes can dominate at different life stages, all of which contribute to an individual’s capacity for behavioral flexibility.

Individual behavioral variation is important for ecological and evolutionary dynamics^30^, and recent advances in animal movement ecology have propelled interest in quantifying the causes and consequences of individual differences in animal movement patterns^31^. Methods to disentangle within-individual variation, such as phenotypically plastic behavioral responses, versus inter-individual variation, such as fixed behavioral traits, offer promising approaches for exploring the ontogeny of behavioral plasticity in heterogeneous populations^32^. For migratory species, flexibility in migration timing contributes to a population’s capacity to adapt to global change^23,33,34^. Consequently, understanding why individuals within the same population differ in their migratory plasticity and ability to track resources is needed not only to advance basic biological knowledge but also to manage populations in the face of anthropogenic environmental change. Our study shows how early life experiences and individual age produce marked intra- and inter-individual differences in phenological plasticity during migration, which may shape a population’s ability to respond to environmental variation and directional global change. Accordingly, these same learning mechanisms could apply to other adaptive components of migration and resource tracking, such as choice of route and selection of seasonal sites.

Finally, understanding the roles of learning and behavioral plasticity in shaping how animals cope with environmental variation has important applications to species conservation in changing landscapes, as managers aim to promote adaptive behaviors^35^. Knowledge about the importance of experiential and social learning is particularly relevant when managers can or must manipulate age and social structures within populations, such as during species reintroductions or assisted migrations^13,36^. In these cases, translocating individuals that are the most likely to learn^37^, or maintaining an optimal ratio of experienced to inexperienced individuals^36^, will be most effective in promoting adaptive behavior. Given that climate change is reshuffling resource phenology across elevational and latitudinal gradients^38,39^, promoting behavioral plasticity in migration phenology will be key to sustaining migration^23^. Our results show that experiential and social learning are crucial for successful resource tracking in a landscape that is affected by both climate change and intensive human land-use, and provides insight into the ontogeny of successful migration.

## Methods

### Crane data

We used data from the Whooping Crane location database from 2002 to 2018, collected in a collaborative effort by the Whooping Crane Eastern Partnership, a public/private partnership dedicated to the reintroduction of the Whooping Crane EMP (www.savingcranes.org). Data collection was performed under consultation with the US Fish and Wildlife Service and complied with all relevant ethical regulations. The Whooping Crane database tracks cranes in the population based on resighting, satellite tracking, or radio-telemetry throughout their lifetime. All birds in the population were uniquely identifiable via colored leg bands and were of known age (i.e., all birds were banded in their first year of life). Birds were located at multiple points during their migration route with telemetry, followed by visual observations of birds on the ground. In some cases, birds with non-functional VHF transmitters were identified via leg bands while in proximity to birds with transmitters. Only in rare exceptions (0.4% of observations) were bird locations identified by telemetry but not visually confirmed.

Training for the aircraft-guided migration consisted of imprinting of birds on costumed humans and ground-based training behind ultralight aircraft at a training facility from just after hatching to approximately 2–3 months of age, followed by training flights behind ultralights for several months on the breeding grounds. In the birds’ first autumn, the ultralight-led migrations themselves began on the breeding grounds in central Wisconsin. These migrations ended at Chassahowitzka National Wildlife Refuge on Florida’s peninsular Gulf Coast from 2001 to 2010; at St. Mark’s National Wildlife Refuge on the Gulf Coast of Florida’s panhandle from 2008 to 2010 and 2012 to 2015; and at Wheeler National Wildlife Refuge in northern Alabama in 2011. Birds remained at each of these wintering sites until they departed of their own accord in the following spring for the northward migration. All subsequent flights in both directions were performed independent of the ultralights. Beginning in 2005, 4 years after the initiation of ultralight-led releases, conspecific-trained birds were released directly on the same breeding grounds in central Wisconsin and were allowed to follow older cranes. Beginning in 2013, conspecific-trained birds were reared by their parents rather than hand-reared by costumed humans prior to release on the breeding grounds. See refs. ^7^^,^^13^ for additional information on the dataset.

During the study period, 105 birds between the ages of 1 and 6 carried ARGOS (*n* = 86) or GPS (*n* = 31) satellite transmitters to more finely track locations. Mean tag duration was 478 days (s.d. 393, range 40–2267 days). All tracks were subsampled to one location fix per day. ARGOS locations were filtered using the Douglas hybrid algorithm implemented in the Movebank tracking database^40^. This method was developed specifically for avian tracking data characterized by periods of range-resident behavior interspersed with periods of rapid and directional movement (e.g., migration). The algorithm filters implausible ARGOS locations based on spatial redundancy, maximum movement rates, and the acuity of tight turning angles which are characteristically created by location estimates with large errors^40^. Positions with an ARGOS location class of 2 or better (error radius <500 m) were always retained. Douglas filtering resulted in an estimated mean ARGOS location error radius <250 m.

Migratory groups were identified via ground observation as the set of individuals that were visually observed together during their migration^7^. Group sizes ranged between 1 and 22 individuals. Each ground-based observation of a crane was assigned a group ID, which was shared with all cranes observed at the same place and time, yielding detailed compositions of known-aged birds for each observation. From this group composition data, the age of the oldest individual(s) in the migratory group (i.e., group age) was extracted for each satellite-tagged bird within each migration season^7^. The age of the oldest bird in a group ranged between 1 year, representing a group consisting only of subadults, to 16 years of age (Supplementary Fig. 1). In the rare instance where a satellite-tagged bird was observed to switch groups (2% of migrations), the age of the oldest bird in any group that a bird was observed with during a given migration season was used for analyses.

### Environmental data

Dynamic environmental variables were extracted for each daily bird location by matching the date and spatial location of the tracking data with those of gridded environmental data. Daily snow depth data on a 1×1 km raster grid were obtained from the National Snow and Ice Data Center Snow Data Assimilation System (SNODAS) (10.7265/N5TB14TC). Eight-day Normalized Difference Vegetation Index (NDVI) data were obtained on a 500 × 500 m raster grid from MODIS via the Environmental Data Automated Track Annotation System (EnvDATA) service on Movebank.org^41^. Point values were extracted from whichever grid cell each tracking location overlaid. To account for interannual variation in winter severity when examining total snow accumulation (see “Analysis”), we used the Accumulated Winter Season Severity Index (AWSSI) obtained from the Midwestern Regional Climate Center which provides an annual index of winter severity based on temperature, snowfall and snow depth (https://mrcc.illinois.edu/research/awssi/indexAwssi.jsp).

### Analysis

We used linear mixed models to test for the influence of environmental variables, training method, age, and group age on daily latitudinal speed during migrations for satellite-tracked birds. The start and end dates of each migration were manually identified for each individual in each year following the methods of Aikens et al.^42^ using time-series of Net Squared Displacement (NSD) (Supplementary Fig. 6). NSD is calculated as the squared distance between an initial location, here the first telemetry position at the breeding site, and subsequent relocations^43^. Migration start and end dates were identified by rapid, extended increases or decreases in NSD^42^ (Supplementary Fig. 6). Models were generated for each migration season (autumn/spring) separately as different factors may shape movement rates during spring and autumn migrations. Snow depth and NDVI were each standardized by *Z*-scoring to facilitate comparison between models. Training method was treated as a categorical variable; all others were treated as numeric. We considered two- and three-way interactions between NDVI/snow and training method, individual age, and group age. We explored inclusion of day-of-year (i.e., ordinal day number between 1 and 366) and its square to control for possible linear and nonlinear effects of time of year unrelated to environmental conditions. Individual, migratory group, and year were modeled as random effects to account for repeated measures, unmodeled heterogeneity, and variation in environmental conditions across years. All numeric fixed effects were first checked for collinearity using Pearson’s correlation coefficient; no variables were discarded because |*r*| < 0.4 for all variables. Spatial autocorrelation was tested for in the model residuals using the R package “ape”^44^; none was detected (Moran’s *I p* value > 0.6). We used AIC model selection to select among a series of candidate models generated from combinations of fixed effects established a priori^45^. We included a model with day-of-year as the only predictor to serve as a null model for baseline comparison^46^.

The generic model with each of the variables was as follows, where “*i*” is individual, “*j*” is year, and “*k*“ is location (see Supplementary Tables 2–4 for all candidate models including interaction terms):

(1) DailyLatSpeed_*i*,*j*,*k*_ = *β*_0_ + *β*_1_*yday_*i*,*j*,*k*_ + *β*_2_*SnowDepth_*i*,*j*,*k*_ + *β*_3_*NDVI_*i*,*j*,*k*_ + *β*_4_*IndividualAge_*i,j*_ + *β*_5_*GroupAge_*i*,*j*_ + *β*_6_[Training_*i*_] + id_*i*_ + group_*i*,*j*_ + year_*j*_ + *ε*_*i*,*j*,*k*_

id_*i*_ ~ Normal(0,*σ*_id_)

group_*i*,*j*_ ~ Normal(0,*σ*_group_)

year_*j*_ ~ Normal(0,*σ*_year_)

*ε*_*i*,*j*,*k*_ ~ Normal(0,*σ*_model_)

Since the reintroduction program began in 2001, the mean latitude of overwintering sites has shifted northward, a behavior termed “shortstopping”^12^. Beginning in 2012, the majority of individuals within the population had adopted shortstopping behavior and the mean latitude of overwintering sites had stabilized (Supplementary Fig. 4). We checked for any confounding factors in our analysis related to shortstopping behavior by re-running our analyses using only data from 2012 onwards, such that any confounding factors due to variability in shortstopping behavior were removed. Results remained consistent for this reduced dataset (Supplementary Fig. 5). Given data deficiency in conspecific-trained birds aged 4–6 years, we also re-ran our analysis for birds only aged 1–3 years; this did not affect results as the effects of sparse data are reflected in the width of the confidence intervals.

We used a linear mixed model to test for the influence of age on cumulative snow exposure during migration, while accounting for interannual variation in winter severity using the Accumulated Winter Season Severity Index. Cumulative snow for each individual in each migration was measured as the sum of the snow depth encountered each day, divided by the number of migration days tracked in order to account for differences in tracking durations. Individual was modeled as a random effect to account for repeated measures. The model formulation was therefore:

(2) CumulativeSnow_*i*,*j*_ = *β*_0_ + *β*_1_*IndividualAge_*i,j*_ + *β*_2_*WinterSeverityIndex_*j*_ + id_*i*_ + *ε*_*i*,*j*_

id_*i*_ ~ Normal(0,*σ*_id_)

ε_*i*,*j*_ ~ Normal(0,*σ*_model_)

Linear mixed models were performed with the “lme4” R package^47^. Partial response curves and confidence intervals were calculated with the “effects” package^48^. All analyses were conducted in R version 4.1.0 (ref. ^49^).

### Reporting summary

Further information on research design is available in the Nature Research Reporting Summary linked to this article.

## Supplementary information

Supplementary Information

Reporting Summary

## Data Availability

The movement data used in this study are available in the Movebank Data Repository following a one-year embargo period after the publication date under accession code 10.5441/001/1.t23vm852 (ref. ^50^).

## Code availability

The code used in analyses is available at the following public repository: https://github.com/briana-abrahms/CraneMigrationSpeed (ref. ^51^).
