# Supplementary material for: Ontogenetic shifts from social to experiential learning drive avian migration timing
Source: Nat Commun. 2021 Dec 16;12:7326. doi: 10.1038/s41467-021-27626-5 (PMC8677782; doi:10.1038/s41467-021-27626-5)
Supplement: Supplementary file 1 — Supplementary Information [file 41467_2021_27626_MOESM1_ESM.pdf]

Supporting Information for

Ontogenetic shifts from social to experiential learning drive avian migration timing

Briana Abrahms, Claire Teitelbaum, Thomas Mueller, and Sarah J. Converse

Correspondence to: abrahms@uw.edu

Table S1. Number of satellite-tracked individuals and location fixes (in parentheses) by training method and age in the eastern migratory whooping crane population.

Age	Ultralight	Conspecific (hand-reared)	Conspecific (parent-reared)
**1**	44 (48854)	24 (119262)	14 (240599)
**2**	26 (24296)	11 (17122)	5 (28610)
**3**	11 (7213)	3 (2151)	2 (1038)
**4**	4 (3514)	2 (1354)	0
**5**	2 (1112)	1 (807)	0
**6**	0	1 (416)	0

Table S2. AIC model selection for autumn migration, subadult (age 1) birds quantifying the effects of intrinsic and extrinsic factors on whooping cranes' latitudinal speeds during migration. SNWZ = snow depth, NDVI = Normalized Difference Vegetation Index, yday = day of year.

formula	deltaAIC
**SNWZ * training* group_age + NDVI * training* group_age + yday + (1|id) + (1|group) + (1|year)**	**0.000**
SNWZ * training + NDVI * training + yday + (1|id) + (1|group) + (1|year)	5.978
SNWZ * training + NDVI * training + group_age + yday + (1|id) + (1|group) + (1|year)	13.384
SNWZ * training + yday + (1|id) + (1|group) + (1|year)	18.247
SNWZ + NDVI + yday + (1|id) + (1|group) + (1|year)	21.683
SNWZ + NDVI + training + group_age + (1|id) + (1|group) + (1|year)	24.121
SNWZ + yday + (1|id) + (1|group) + (1|year)	24.557
SNWZ * group_age + yday + (1|id) + (1|group) + (1|year)	31.078
SNWZ + NDVI + training + group_age + yday + (1|id) + (1|group) + (1|year)	34.361
SNWZ * group_age + NDVI * group_age + training + yday + (1|id) + (1|group) + (1|year)	36.068
SNWZ + NDVI + training + group_age + I(yday^2) + (1|id) + (1|group) + (1|year)	46.728
SNWZ + NDVI + training + yday + I(yday^2) + (1|id) + (1|group) + (1|year)	47.510
SNWZ + NDVI + group_age + yday + I(yday^2) + (1|id) + (1|group) + (1|year)	49.039
SNWZ + NDVI + training + group_age + yday + I(yday^2) + (1|id) + (1|group) + (1|year)	54.546
SNWZ + training + group_age + yday + I(yday^2) + (1|id) + (1|group) + (1|year)	57.567
NDVI * training + yday + (1|id) + (1|group) + (1|year)	192.687
NDVI + yday + (1|id) + (1|group) + (1|year)	197.608
yday + (1|id) + (1|group) + (1|year)	206.153
NDVI * group_age + yday + (1|id) + (1|group) + (1|year)	207.878
NDVI + training + group_age + yday + I(yday^2) + (1|id) + (1|group) + (1|year)	231.154

Table S3. AIC model selection for autumn migration, all ages quantifying the effects of intrinsic and extrinsic factors on whooping cranes' latitudinal speeds during migration. SNWZ = snow depth, NDVI = Normalized Difference Vegetation Index, yday = day of year.

**formula**	**deltaAIC**
**SNWZ * training* age + NDVI * training* age + yday + (1|id) + (1|group) + (1|year)**	**0.000**
SNWZ * training* age + NDVI * training* age + group_age + yday + (1|id) + (1|group) + (1|year)	5.049
SNWZ * training + SNWZ * age + NDVI * training + NDVI * age + yday + (1|id) + (1|group) + (1|year)	6.816
SNWZ * training* group_age + NDVI * training* group_age + yday + (1|id) + (1|group) + (1|year)	7.161
SNWZ * training* group_age + NDVI * training* group_age + age + yday + (1|id) + (1|group) + (1|year)	11.928
SNWZ * training + NDVI * training + age + group_age + yday + (1|id) + (1|group) + (1|year)	12.924
SNWZ * training* age + yday + (1|id) + (1|group) + (1|year)	20.089
SNWZ + NDVI + yday + (1|id) + (1|group) + (1|year)	23.122
SNWZ + yday + (1|id) + (1|group) + (1|year)	25.488
SNWZ * age + yday + (1|id) + (1|group) + (1|year)	28.354
SNWZ * training + yday + (1|id) + (1|group) + (1|year)	28.354
SNWZ + NDVI + training + age + group_age + (1|id) + (1|group) + (1|year)	29.248
SNWZ * age + NDVI * age + training + group_age + yday + (1|id) + (1|group) + (1|year)	38.067
SNWZ * age* group_age + NDVI * age* group_age + yday + (1|id) + (1|group) + (1|year)	39.071
SNWZ + NDVI + training + age + group_age + yday + (1|id) + (1|group) + (1|year)	39.196
SNWZ * group_age + NDVI * group_age + training + age + yday + (1|id) + (1|group) + (1|year)	41.447
SNWZ + NDVI + training + age + group_age + I(yday^2) + (1|id) + (1|group) + (1|year)	51.683
SNWZ + NDVI + age + group_age + yday + I(yday^2) + (1|id) + (1|group) + (1|year)	53.618
SNWZ + NDVI + training + age + yday + I(yday^2) + (1|id) + (1|group) + (1|year)	55.274
SNWZ + NDVI + training + group_age + yday + I(yday^2) + (1|id) + (1|group) + (1|year)	55.495
SNWZ + NDVI + training + age + group_age + yday + I(yday^2) + (1|id) + (1|group) + (1|year)	59.938
SNWZ + training + age + group_age + yday + I(yday^2) + (1|id) + (1|group) + (1|year)	63.006
NDVI * training* age + yday + (1|id) + (1|group) + (1|year)	205.337
NDVI + yday + (1|id) + (1|group) + (1|year)	208.842
NDVI * age + yday + (1|id) + (1|group) + (1|year)	215.089
NDVI * training + yday + (1|id) + (1|group) + (1|year)	215.089
yday + (1|id) + (1|group) + (1|year)	216.990

Table S4. AIC model selection for spring migration, all ages quantifying the effects of intrinsic and extrinsic factors on whooping cranes' latitudinal speeds during migration. SNWZ = snow depth, NDVI = Normalized Difference Vegetation Index, yday = day of year.

**formula**	**deltaAIC**
**SNWZ * training* age + NDVI * training* age + yday + (1|id) + (1|group) + (1|year)**	**0.000**
SNWZ * training + SNWZ * age + NDVI * training + NDVI * age + yday + (1|id) + (1|group) + (1|year)	2.477
SNWZ + NDVI + yday + (1|id) + (1|group) + (1|year)	3.515
SNWZ * training* age + NDVI * training* age + group_age + yday + (1|id) + (1|group) + (1|year)	5.042
SNWZ * training + NDVI * training + age + group_age + yday + (1|id) + (1|group) + (1|year)	9.986
SNWZ * age + NDVI * age + training + group_age + yday + (1|id) + (1|group) + (1|year)	16.917
SNWZ + NDVI + training + age + group_age + yday + (1|id) + (1|group) + (1|year)	19.164
SNWZ + NDVI + training + age + group_age + (1|id) + (1|group) + (1|year)	23.329
SNWZ + NDVI + training + age + group_age + I(yday^2) + (1|id) + (1|group) + (1|year)	23.905
SNWZ + NDVI + training + group_age + yday + I(yday^2) + (1|id) + (1|group) + (1|year)	24.540
SNWZ + NDVI + age + group_age + yday + I(yday^2) + (1|id) + (1|group) + (1|year)	25.308
SNWZ + NDVI + training + age + yday + I(yday^2) + (1|id) + (1|group) + (1|year)	25.435
SNWZ * group_age + NDVI * group_age + training + age + yday + (1|id) + (1|group) + (1|year)	28.496
SNWZ + NDVI + training + age + group_age + yday + I(yday^2) + (1|id) + (1|group) + (1|year)	30.753
SNWZ * training* group_age + NDVI * training* group_age + yday + (1|id) + (1|group) + (1|year)	38.894
SNWZ * training* group_age + NDVI * training* group_age + age + yday + (1|id) + (1|group) + (1|year)	44.938
SNWZ + yday + (1|id) + (1|group) + (1|year)	80.041
SNWZ * training + yday + (1|id) + (1|group) + (1|year)	81.838
SNWZ * training* age + yday + (1|id) + (1|group) + (1|year)	83.069
SNWZ * age + yday + (1|id) + (1|group) + (1|year)	86.473
SNWZ + training + age + group_age + yday + I(yday^2) + (1|id) + (1|group) + (1|year)	108.792
NDVI * training + yday + (1|id) + (1|group) + (1|year)	420.473
NDVI + yday + (1|id) + (1|group) + (1|year)	423.706
NDVI * age + yday + (1|id) + (1|group) + (1|year)	428.011
NDVI * training* age + yday + (1|id) + (1|group) + (1|year)	429.920
NDVI + training + age + group_age + yday + I(yday^2) + (1|id) + (1|group) + (1|year)	448.508
yday + (1|id) + (1|group) + (1|year)	572.979

Table S5. Random effect variances for top-ranked models (variance ± standard deviation) quantifying the effects of intrinsic and extrinsic factors on whooping cranes' latitudinal speeds during migration. Individual ID is the random effect of each bird's individual identity; Group ID is the random effect of each bird's migratory group.

**Model**	**Individual ID**	**Group ID**	**Year**	**Residual**
Autumn, subadult	0.71 ± 0.84	$1.3*10^{-9}$ ± $3.6*10^{-5}$	0.13 ± 0.36	7.61 ± 2.76
Spring, all ages	0.22 ± 0.46	0.27 ± 0.52	0.0 ± 0.0	4.53 ± 2.13
Autumn, all ages	0.37 ± 0.61	0.97 ± 0.98	0.0 ± 0.0	4.32 ± 2.08

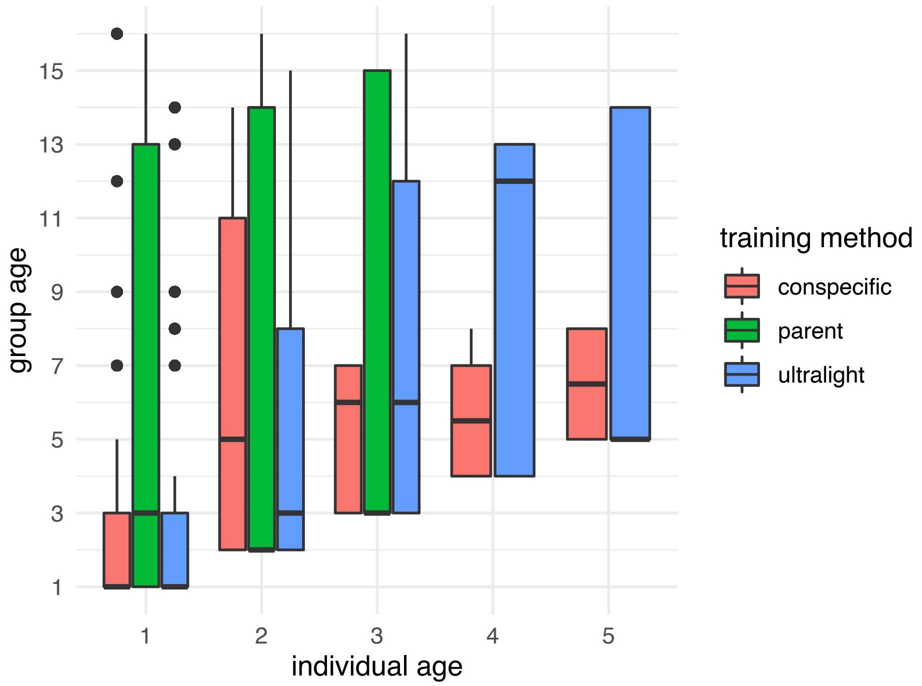

Figure S1. Boxplot of the age of the oldest bird in a migratory bird (i.e., ‘group age’) by age and training method of satellite-tracked birds used in analyses (*n* = 105 individuals, 242 migrations). Thick line in boxplots represents the median (50th percentile) summary statistic; upper and lower bounds of box represent 75th and 25th percentiles, respectively. Black dots represent outliers.

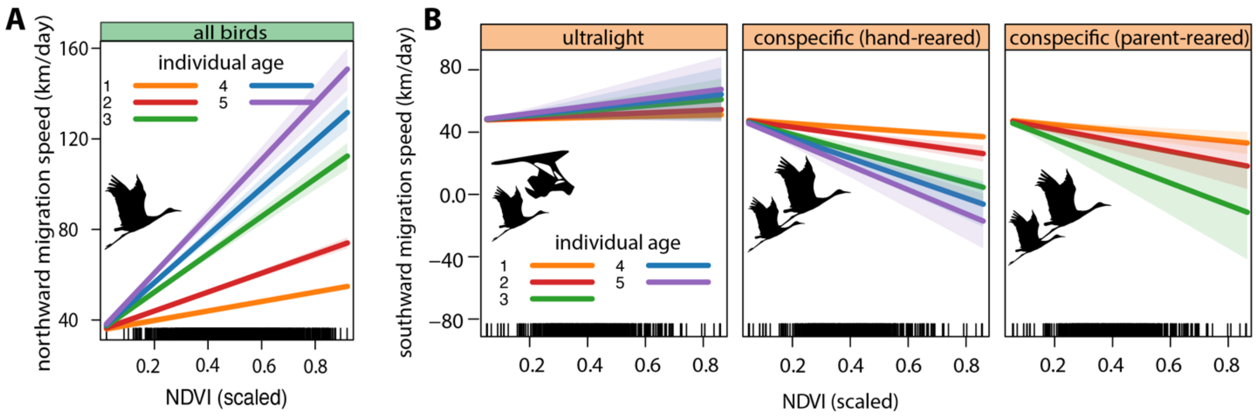

Figure S2. Response lines for interactions between individual age and NDVI on latitudinal speed during (a) spring and (b) autumn migrations. Shading represents 95% confidence intervals.

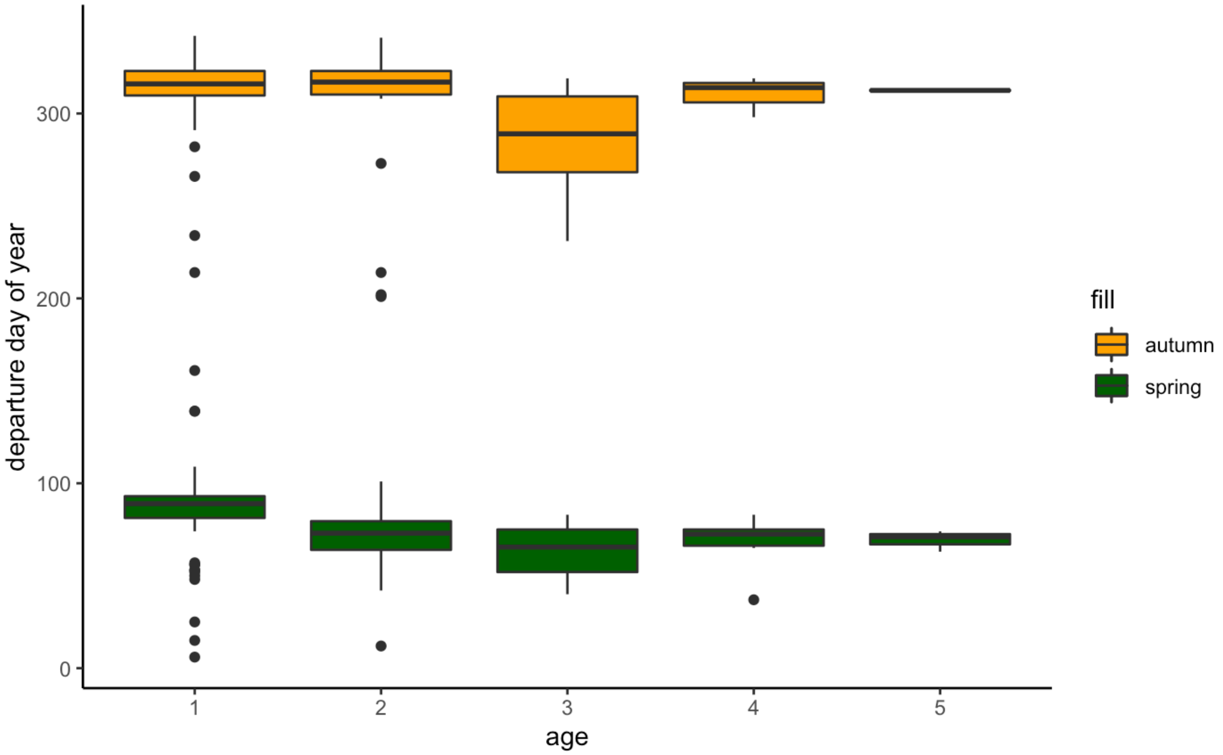

Figure S3. Departure dates at the start of each migration season by individual age (*n* = 105 individuals, 242 migrations). Linear mixed effects regression for departure day ~ age in spring ß=-7.15 days, 95% CI = -10.18 - -4.13 days. Age did not have a significant effect on departure dates in autumn. Thick line in boxplots represents the median (50th percentile) summary statistic; upper and lower bounds of box represent 75th and 25th percentiles, respectively. Black dots represent outliers.

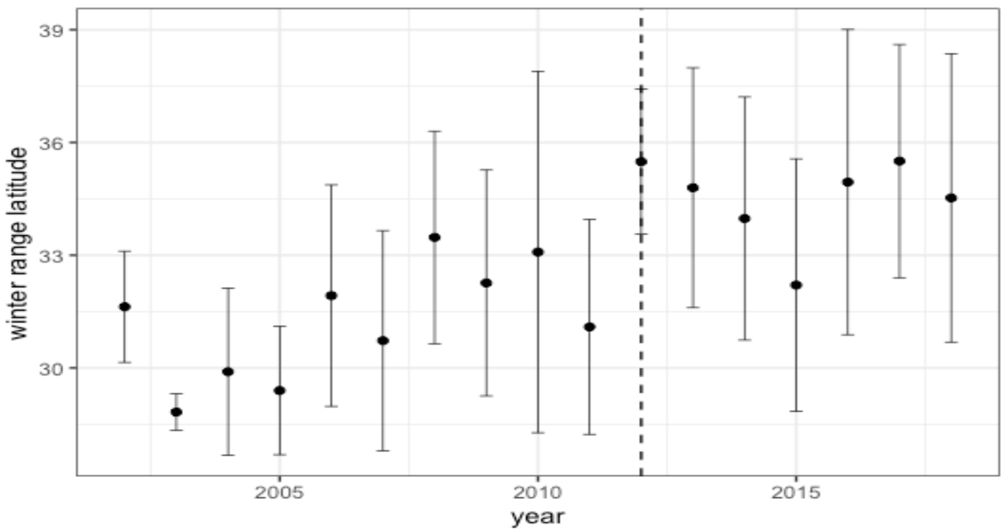

Figure S4. Mean +/- standard deviation of the eastern migratory whooping crane population's winter range latitudes over time (*n* = 105 individuals, 242 migrations). The dashed line in 2012 shows the beginning of the recent period where the locations of overwintering sites has stabilized, and for which we re-ran our analyses.

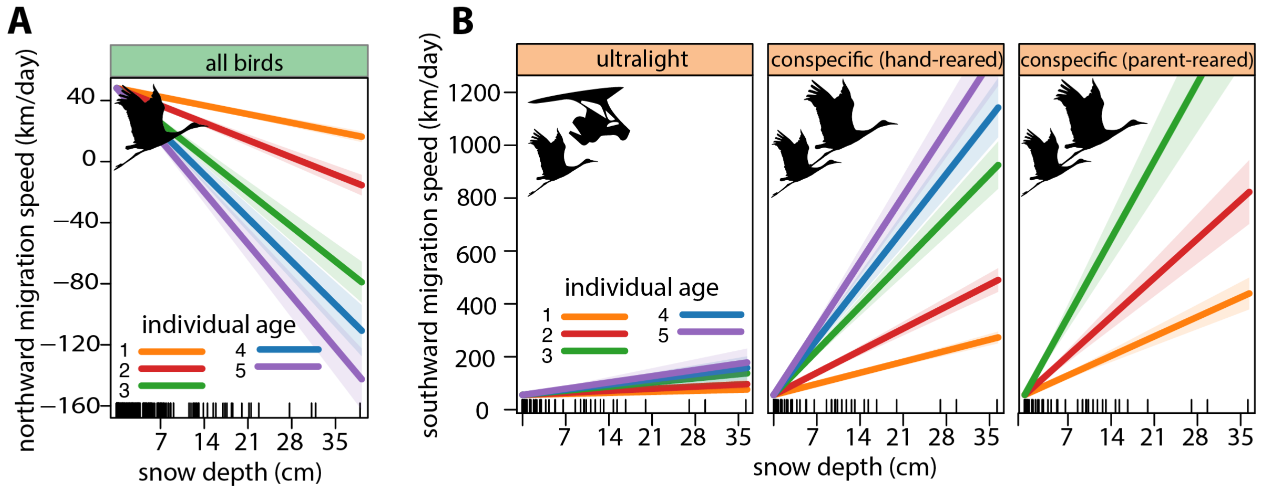

Figure S5. Response lines for interactions between individual age and snow depth on latitudinal speed during (a) spring and (b) autumn migrations using data from 2012 onwards. Shading represents 95% confidence intervals.

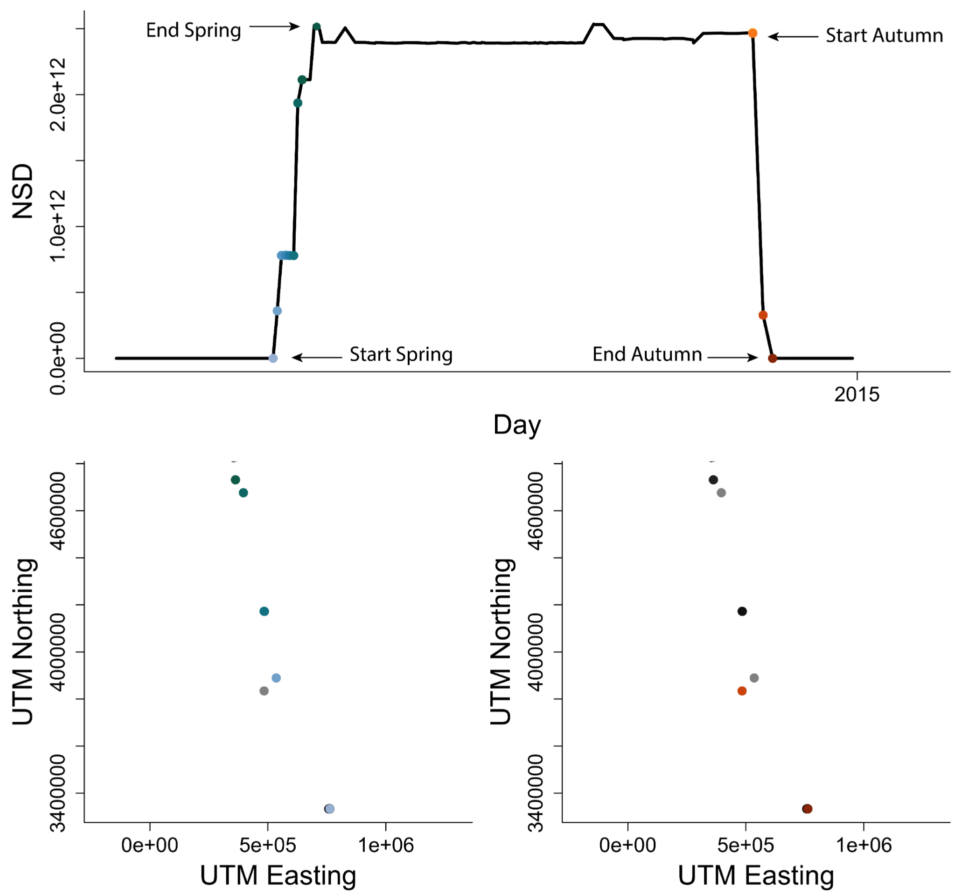

Figure S6. An example of a typical time series of Net Squared Displacement (NSD) over a crane's migration cycle (top), with maps of locations highlighting spring (bottom left) and autumn (bottom right) migration corridors. Method adapted from Aikens et al. 2017.
