# Supplementary material for: Ontogenetic shifts from social to experiential learning drive avian migration timing
Source: Nat Commun. 2021 Dec 16;12:7326. doi: 10.1038/s41467-021-27626-5 (PMC8677782; doi:10.1038/s41467-021-27626-5)
Supplement: Supplementary file 2 — Reporting Summary [file 41467_2021_27626_MOESM2_ESM.pdf]

nature portfolio

Corresponding author(s): Briana Abrahms

Last updated by author(s): Nov 22, 2021

Reporting Summary

Nature Portfolio wishes to improve the reproducibility of the work that we publish. This form provides structure for consistency and transparency in reporting. For further information on Nature Portfolio policies, see our Editorial Policies and the Editorial Policy Checklist.

Statistics

For all statistical analyses, confirm that the following items are present in the figure legend, table legend, main text, or Methods section.

n/a	Confirmed	
☐	☒	The exact sample size (*n*) for each experimental group/condition, given as a discrete number and unit of measurement
☐	☒	A statement on whether measurements were taken from distinct samples or whether the same sample was measured repeatedly
☐	☒	The statistical test(s) used AND whether they are one- or two-sided<br>*Only common tests should be described solely by name; describe more complex techniques in the Methods section.*
☐	☒	A description of all covariates tested
☐	☒	A description of any assumptions or corrections, such as tests of normality and adjustment for multiple comparisons
☐	☒	A full description of the statistical parameters including central tendency (e.g. means) or other basic estimates (e.g. regression coefficient) AND variation (e.g. standard deviation) or associated estimates of uncertainty (e.g. confidence intervals)
☐	☒	For null hypothesis testing, the test statistic (e.g. *F*, *t*, *r*) with confidence intervals, effect sizes, degrees of freedom and *P* value noted<br>*Give P values as exact values whenever suitable.*
☒	☐	For Bayesian analysis, information on the choice of priors and Markov chain Monte Carlo settings
☐	☒	For hierarchical and complex designs, identification of the appropriate level for tests and full reporting of outcomes
☐	☒	Estimates of effect sizes (e.g. Cohen's *d*, Pearson's *r*), indicating how they were calculated

Our web collection on statistics for biologists contains articles on many of the points above.

Software and code

Policy information about availability of computer code

Data collection	This is a field study. No software was used in data collection.
Data analysis	Data were analyzed in R Programming Language version 4.1.0. Linear mixed models were performed with the 'lme4' R package. Partial response curves and confidence intervals were calculated with the 'effects' package. The code used in analyses is available at the following public repository: https://github.com/briana-abrahms/CraneMigrationSpeed (DOI: 10.5281/zenodo.5719357).

For manuscripts utilizing custom algorithms or software that are central to the research but not yet described in published literature, software must be made available to editors and reviewers. We strongly encourage code deposition in a community repository (e.g. GitHub). See the Nature Portfolio guidelines for submitting code & software for further information.

Data

Policy information about availability of data

All manuscripts must include a data availability statement. This statement should provide the following information, where applicable:

- Accession codes, unique identifiers, or web links for publicly available datasets
- A description of any restrictions on data availability
- For clinical datasets or third party data, please ensure that the statement adheres to our policy

We used data from the Whooping Crane location database from 2002 to 2018, collected in a collaborative effort by the Whooping Crane Eastern Partnership, a public/private partnership dedicated to the reintroduction of the Whooping Crane eastern migratory population (www.savingcranes.org). The movement data analyzed in the current study will be made publicly available in the Movebank Data Repository, https://doi.org/10.5441/001/1.t23vm852, following a 1-year embargo period from the publication date.

Field-specific reporting

Please select the one below that is the best fit for your research. If you are not sure, read the appropriate sections before making your selection.

☐ Life sciences ☐ Behavioural & social sciences ☒ Ecological, evolutionary & environmental sciences

For a reference copy of the document with all sections, see nature.com/documents/nr-reporting-summary-flat.pdf

Ecological, evolutionary & environmental sciences study design

All studies must disclose on these points even when the disclosure is negative.

Study description	We built linear mixed models using satellite-tracking data from whooping crane migrations to test for the effect of environmental conditions, individual experience, and social context on migration timing.
Research sample	The research sample is 105 satellite-tagged whooping cranes (Grus americana; 84 female, 21 male) from the reintroduced eastern migratory whooping crane population, aged 1-6, between 2002 and 2018. The sample is meant to represent the movement patterns of the population, which includes a total of 504 birds released within the study period. Candidate birds were selected for tagging based on a health assessment of body condition to migrate with extra tag weight; beyond this birds were selected to represent the range of rearing/training methods used in the population.
Sampling strategy	This is a field study to monitor reintroduced whooping cranes. Data were collected and supplied by the Eastern Whooping Crane Partnership (www.savingcranes.org). Sample size was determined by the number of available individuals in the population being released. Birds < 6 years of age were targeted for satellite tracking to monitor the establishment of migratory patterns following initial release. 105 total individuals were satellite-tracked; this represents ~20% of the total population and is a large sample size in the field of animal movement ecology, which regularly publishes papers with sample sizes < 30 individuals. Satellite tracking devices collected data for as long as batteries lasted, on average 478 days (sd 393, range 40-2267 days), for a total of >500,000 GPS locations collected.
Data collection	We used data from the Whooping Crane location database from 2002 to 2018, collected in a collaborative effort by the Whooping Crane Eastern Partnership, a public/private partnership dedicated to the reintroduction of the Whooping Crane eastern migratory population (www.savingcranes.org). This database tracks cranes in the population based on resighting, satellite tracking, or radio-telemetry. All birds in the population were uniquely identifiable via colored leg bands and were of known age. Birds were located at multiple points during their migration route with telemetry, followed by visual observations of birds on the ground. In some cases, birds with non-functional VHF transmitters were identified via leg bands while in proximity to birds with transmitters. Only in rare exceptions were bird locations identified by telemetry but not visually confirmed. Satellite tracking data on locations of birds were downloaded directly and in most cases were used to locate birds. Birds were also located using triangulation of radio telemetry signals. When birds were located (by multiple different field biologists over the course of the study), data were recorded on the identity of birds present based on leg bands. Data were collected on either paper or electronic data sheets. During the study period, 105 birds carried ARGOS (n=86) or GPS (n=31) satellite transmitters to more finely track locations.
Timing and spatial scale	Tracking data were collected continuously between 2002 and 2018 over the coarse of the reintroduction program in order to monitor the emergence of migration patterns following release, as determined in the reintroduction protocol. Tracking data covered ~2000 km between seasonal ranges in Wisconsin and Florida, USA to collect data over the entire migration path.
Data exclusions	Movement data from birds aged 0-1 (i.e. juvenile) were excluded to focus on subadult (age 1) and adult (> age 1) migration patterns.
Reproducibility	Not applicable as this is an observational field study; no experiments or manipulations were conducted.
Randomization	In linear mixed models with daily latitudinal speed as the response variable, individual, migratory group, and year were modeled as random effects to account for repeated measures.
Blinding	Not applicable as this is an observational field study; no blinding during data collection or analysis occurred

Did the study involve field work? ☒ Yes ☐ No

Field work, collection and transport

Field conditions	Bird release occurred in late summer and early autumn in Wisconsin prior to birds' first autumn migration to wintering grounds following reintroduction. Data were collected and supplied by the Eastern Whooping Crane Partnership (www.savingcranes.org). Field conditions spanned the year-round weather conditions existing between Wisconsin and Florida, including occurrence of snow at higher latitudes in late fall/winter/early spring and onset of warm weather at lower latitudes in the late spring/summer.
Location	Tagging occurred during bird release into breeding grounds in central Wisconsin (43.87° N, - 89.23° E).
Access & import/export	Data collection was performed in partnership with the US Fish and Wildlife Service as part of the Eastern Whooping Crane Partnership. No import/export of samples occurred.
Disturbance	The study was part of a monitoring effort of individuals post-reintroduction. Data were collected and supplied by the Eastern Whooping Crane Partnership (www.savingcranes.org). Tagging devices weighed <5% of individual body mass to minimize any possible

deleterious effects of wear a satellite tracking device per field standard. Birds in the study were raised in captivity and so were accustomed to interacting with handlers. Costumes were used by handlers to mask the human form and thereby minimize habituation. Captive management practices for whooping cranes are established in the literature, and were followed rigorously. Bands and tracking devices were added to birds prior to release. In some cases, birds whose tracking devices failed were recaptured by costumed handlers to replace tracking devices. Protocols to minimize handling time and maximize bird safety were followed. During observation of birds in the field, field biologists maintained a distance from birds and ensured that birds were engaging in normal behaviors during observation.

Reporting for specific materials, systems and methods

We require information from authors about some types of materials, experimental systems and methods used in many studies. Here, indicate whether each material, system or method listed is relevant to your study. If you are not sure if a list item applies to your research, read the appropriate section before selecting a response.

Materials & experimental systems

n/a	Involved in the study
☒	☐ Antibodies
☒	☐ Eukaryotic cell lines
☒	☐ Palaeontology and archaeology
☐	☒ Animals and other organisms
☒	☐ Human research participants
☒	☐ Clinical data
☒	☐ Dual use research of concern

Methods

n/a	Involved in the study
☒	☐ ChIP-seq
☒	☐ Flow cytometry
☒	☐ MRI-based neuroimaging

Animals and other organisms

Policy information about studies involving animals; ARRIVE guidelines recommended for reporting animal research

Laboratory animals	The study did not involve laboratory animals.
Wild animals	105 whooping cranes (Grus americana; 84 female, 21 male) aged 1-6 were banded and fitted with satellite tracking devices. All birds were reared in captivity and tagged upon release as part of a reintroduction effort. No birds were harmed during this process. Data were collected and supplied by the Eastern Whooping Crane Partnership (www.savingcranes.org).
Field-collected samples	No field-collected samples were used in the study.
Ethics oversight	Data were collected and supplied by the Eastern Whooping Crane Partnership (www.savingcranes.org) under consultation with the US Fish and Wildlife Service.

Note that full information on the approval of the study protocol must also be provided in the manuscript.
